# Double-stranded sperm DNA fragmentation measured with neutral comet assay as a predictor of IVF outcomes: evidence from three European clinics in a multi-centred prospective study

**DOI:** 10.1093/humrep/deag046

**Published:** 2026-03-28

**Authors:** Peter Humaidan, Betina B Povlsen, Andrew J Drakeley, Mette B Jensen, Anette V Gabrielsen, Sara H McDowell, Luca Moore, Craig J Ledgerwood, Lewis Rae, Alastair Sloan, Martin Lawlor, Lynsey Poots, Elizabeth Bailie, Rebecca L Lunt, Emily Newton, Rachel C Gregoire, Tara C B Moore, Sandro C Esteves

**Affiliations:** Department of Clinical Medicine, Aarhus University, Aarhus, Denmark; Central Denmark Region, University Clinic for Fertility, Skive, Denmark; Central Denmark Region, University Clinic for Fertility, Skive, Denmark; Hewitt Fertility Centre, Liverpool Women’s NHS Foundation Trust, Liverpool, UK; University of Liverpool, Liverpool, UK; Central Denmark Region, University Clinic for Fertility, Skive, Denmark; The Fertility Clinic, Horsens Regional Hospital, Horsens, Denmark; Future Medicines Institute, Centre for Digital Health Technology, Ulster University, Belfast, UK; Department of Biology, University of York, York, UK; Examen, Weavers Court Business Park, Belfast, UK; Examen, Weavers Court Business Park, Belfast, UK; Examen, Weavers Court Business Park, Belfast, UK; Examen, Weavers Court Business Park, Belfast, UK; Examen, Weavers Court Business Park, Belfast, UK; Future Medicines Institute, Centre for Digital Health Technology, Ulster University, Belfast, UK; Hewitt Fertility Centre, Liverpool Women’s NHS Foundation Trust, Liverpool, UK; Hewitt Fertility Centre, Liverpool Women’s NHS Foundation Trust, Liverpool, UK; Hewitt Fertility Centre, Liverpool Women’s NHS Foundation Trust, Liverpool, UK; Future Medicines Institute, Centre for Digital Health Technology, Ulster University, Belfast, UK; Examen, Weavers Court Business Park, Belfast, UK; Department of Clinical Medicine, Aarhus University, Aarhus, Denmark; ANDROFERT, Andrology and Human Reproduction Clinic, Campinas, Brazil; Division of Urology, Department of Surgery, University of Campinas (UNICAMP), Campinas, Brazil

**Keywords:** *in vitro* fertilization, sperm dsDNA fragmentation, comet assay, live birth, miscarriage

## Abstract

**STUDY QUESTION:**

Can measurement of double-stranded sperm DNA fragmentation (dsSDF) via a neutral comet assay predict the probability of live birth following IVF?

**SUMMARY ANSWER:**

In a multicentre IVF cohort, dsSDF measured by a neutral comet assay was a strong, independent predictor of live birth.

**WHAT IS KNOWN ALREADY:**

While much of the focus has traditionally been on female factors, emerging research highlights sperm DNA fragmentation as a significant contributor to reproductive outcomes. Over the past decade, studies have shown that different types of sperm DNA damage can affect reproduction differently, with single-stranded breaks being closely linked to reduced spontaneous conception rates, while double-stranded breaks are linked to higher miscarriage rates.

**STUDY DESIGN, SIZE, DURATION:**

Prospective cohort study including a total of 302 males from three European IVF clinics, over a 3-year study period (March 2021–October 2024), with 126 healthy sperm donors with confirmed live birth serving as controls.

**PARTICIPANTS/MATERIALS, SETTING, METHODS:**

dsSDF was quantified with a neutral comet assay, expressed as Average Comet Score (ACS) and Incidence of Damage (IOD). The primary outcome was live birth per initiated cycle. Associations were evaluated using multivariable logistic regression, adjusting for female and male age (and centre in sensitivity analyses).

**MAIN RESULTS AND THE ROLE OF CHANCE:**

Across the cohort, 30% of couples achieved a live birth. Higher dsSDF was associated with reduced odds of live birth, and this association remained statistically significant after adjustment for female age, male age, and recruitment site. Both ACS and IOD were independently predictive of live birth in adjusted models. For ACS, each 1-point increase was associated with 16% lower odds of live birth (OR = 0.84, 95% CI 0.72–0.97; *P* = 0.026). For IOD, each 1-point increase corresponded to 5% lower odds of live birth (OR = 0.95, 95% CI 0.90–0.99; *P* = 0.025). As expected, female age remained a strong inverse predictor of live birth across models (OR = 0.86, 95% CI 0.78–0.94; *P* < 0.001). Using a pragmatic threshold of IOD ≥ 6%, couples were identified with approximately half the odds of achieving a live birth compared to those with IOD < 6% at similar female ages (OR = 0.51, 95% CI 0.28–0.94; *P* = 0.029). The adverse association between dsSDF and live birth was stronger at higher female ages.

**LIMITATIONS, REASONS FOR CAUTION:**

This study examined couples undergoing their first or only IVF cycle and did not include couples with repeat IVF failures. Limitations include potential centre-level confounding, which may benefit from mixed-effects modelling. We did not collect or adjust for several cycle-level covariates that influence live birth (e.g. IVF vs ICSI, number of oocytes retrieved, embryo transfer strategy, use of preimplantation genetic testing for aneuploidy, stimulation protocol), so residual confounding is possible.

**WIDER IMPLICATIONS OF THE FINDINGS:**

These results support dsSDF as a clinically relevant biomarker that complements conventional semen parameters.

**STUDY FUNDING/COMPETING INTEREST(S):**

The study was part-funded using an unrestricted medical educational grant provided by Merck Serono Limited (0111897641) to the Liverpool Women’s Hospital. T.C.B.M., S.H.M., and E.B. were funded in part by UKRI SIP FMI and Peace Plus HF-TIC grants with Ulster University. L.R., A.S., M.L., C.J.L., L.P., and T.C.B.M. are employed at Examen Lab LTD. A.J.D. is a recipient of Merck Serono Limited (0111897641) grant to the Liverpool Women’s Hospital. P.H. has received unrestricted research grants from Merck and Gedeon Richter Nordics and honoraria for lectures from Merck, Gedeon Richter, and IBSA. The remaining authors have nothing to disclose.

**TRIAL REGISTRATION NUMBER:**

N/A.

## Introduction

Traditional semen analysis, encompassing sperm concentration, motility, and morphology, remains the cornerstone of male fertility laboratory assessment. However, these parameters often fail to account for the functional integrity of the sperm genome (Agarwal *et al*., 2003; Zini and Sigman, 2009; Esteves, 2022). In line with this, sperm DNA fragmentation (SDF) has emerged as an informative biomarker of the male reproductive potential. SDF refers to the presence of single- or double-stranded breaks in the sperm genome, which are generated through various non-mutually exclusive mechanisms (Ribas-Maynou *et al*., 2012; Esteves *et al.*, 2021; Conti *et al.*, 2024) and can be transmitted to the zygote due to the inability of mature sperm to repair genotoxic damage (De Iuliis *et al.*, 2009; Smith *et al*., 2013; Champroux *et al.*, 2016).

Double-stranded sperm DNA fragmentation (dsSDF) can arise from several non-mutually exclusive pathways, such as oxidative stress-induced DNA cleavage, in which reactive oxygen species overwhelm antioxidant defences, leading to strand scission (Agarwal *et al*., 2020). Additional sources of dsSDF include apoptosis during spermatogenesis, defective chromatin remodelling during protamination, and environmental/genotoxic exposures (Shamsi *et al*., 2011; Simon *et al.*, 2017a; Agarwal *et al.*, 2020). Unlike single-strand breaks, which may be transient or reparable by the oocyte following fertilization, dsSDF usually represent irreversible damage due to the inability of the oocyte repair machinery to mount an effective DNA repair response (Smith *et al*., 2013; Aitken *et al*., 2014).

While attention previously focused on female factors with regard to infertility, recent research points towards the level of DNA fragmentation present in the sperm as an important contributor to the reproductive outcome (Esteves *et al.*, 2021; Nielsen *et al.*, 2023). Over the last decade, it has been demonstrated that different types of DNA damage are associated with varying reproductive consequences, establishing that the presence of single-stranded breaks may cause a reduction in natural conception rates, whereas a high incidence of double-stranded breaks in sperm can cause an increase in miscarriage rates (Ribas-Maynou *et al*., 2012; Nielsen *et al.*, 2023; Drakeley *et al.*, 2026). Systematic reviews and meta-analyses of SDF to date have acknowledged the heterogeneous and variable quality of the available data, as well as the presence of potential bias related to either the SDF testing method or single motile sperm selection in ICSI (Ribas-Maynou *et al*., 2021; Nielsen *et al.*, 2023). A growing body of evidence now suggests that recurrent miscarriage may have a male component, as the male gamete contributes one-half of the genomic content to the embryo (Zhao *et al.*, 2014; Simon *et al.*, 2017a; ESHRE Guideline Group on RPL *et al*., 2023; Deng *et al.*, 2019; Naglot *et al*., 2023). This means that SDF testing could be a method of predicting a couple’s likelihood of experiencing recurrent pregnancy loss (RPL) (Drakeley *et al.*, 2026).

Despite mounting evidence of the male contribution to ART failure and RPL, the clinical integration of dsSDF testing remains limited, partly due to variability in study designs and assay standardization (Agarwal *et al.*, 2016). However, recent guidelines suggest that males in couples with unexplained infertility, repeat IVF failure, or RPL should be evaluated for SDF (Esteves *et al.*, 2021; WHO, 2021), particularly using methods that capture dsSDF.

Assays commonly used to assess SDF, including the sperm chromatin structure assay, the sperm chromatin dispersion test, and the TUNEL assay, primarily provide measures of global DNA fragmentation and do not specifically distinguish between single- and double-strand DNA breaks when applied in routine clinical or research settings (Evenson *et al*., 2002; Fernández *et al*., 2005; Esteves *et al*., 2021). In contrast, the comet assay performed under neutral conditions preferentially detects double-strand DNA breaks, thereby offering greater specificity for this form of damage (Ribas-Maynou and Benet, 2019). Importantly, accumulating evidence indicates that elevated dsSDF is more strongly associated with RPL than other forms of sperm DNA damage (Ribas-Maynou *et al*., 2012; Drakeley *et al*., 2026). Accordingly, dsSDF should be viewed as a complementary biomarker that may provide additional biological insight rather than as a replacement for established SDF assays.

It is a well-known fact that female age is a critical determinant of live birth in IVF, with advancing maternal age associated with a progressive decline in oocyte quality, embryo competence, and implantation potential, ultimately leading to lower live birth rates. While oocytes possess intrinsic DNA repair mechanisms capable of correcting sperm DNA damage, including double-stranded breaks, this capacity diminishes with increasing maternal age, potentially reducing the embryo’s ability to overcome high levels of dsSDF and negatively impacting fertilization, embryo development, and live birth (Zenzes, 2000; Aitken and De Iuliis, 2010; Simon *et al*., 2017a). These findings underscore the importance of minimizing SDF in IVF cycles involving older female partners, where oocyte-mediated DNA repair may be insufficient (Esteves *et al.*, 2020; Lira Neto *et al.*, 2021; Humaidan *et al.*, 2022; Kobayashi and Imanaka, 2024; Sharma *et al*., 2024). Recent reviews highlight that, although the neutral comet assay effectively detects dsSDF, its direct association with IVF success, especially in the context of advancing female age, remains poorly established (Simon *et al*., 2017b; Ribas-Maynou and Benet, 2019; Adler *et al*., 2023). Thus, given that oocyte DNA repair capacity declines with maternal age (Zenzes, 2000; Aitken and De Iuliis, 2010), understanding how dsSDF impacts fertilization and embryo development across different age groups is essential.

We previously reported the use of the Extend^®^ neutral comet assay to quantify dsSDF in a large cohort of males from couples attending a recurrent miscarriage clinic, demonstrating a strong association between dsSDF and miscarriage attributed to male factors (Drakeley *et al*., 2026). The neutral comet assay produces two complementary metrics: Average Comet Score (ACS), which reflects the mean percentage of DNA migrating into the comet tail across all analysed sperm (a continuous measure of overall damage burden), and Incidence of Damage (IOD), which represents the proportion of sperm exceeding a predefined threshold of %Tail DNA (a measure of the frequency of highly damaged cells).

Whether these dsSDF metrics predict live birth after IVF across diverse clinical settings, and how this interacts with female age, remains less well established. In this prospective multicentre cohort of males undergoing IVF across three European clinics, we measured dsSDF using the Extend^®^ neutral comet assay and assessed the relationship between ACS and IOD and subsequent live birth, adjusting for parental age and centre. We further explored the clinical utility of a pragmatic IOD cut-off and developed an interpretable probability framework integrating female age and dsSDF to support patient counselling.

## Materials and methods

### Ethical considerations

This study was a prospective, multicentre cohort study conducted at three European IVF clinics: Hewitt Fertility Centre, Liverpool Women’s NHS Foundation Trust (UK), Skive Fertility Clinic, and Horsens Fertility Clinic (both Denmark), in collaboration with Examen Laboratories (Belfast, UK) over a 3-year study period (March 2021–October 2024). Ethical approval was granted by the Wales Research Ethics Committee 4 Wrexham (IRAS: 263828) for the UK arm. Ethical protocols were also adhered to at Skive and Horsens (The Ethics Committee for Central Denmark Region case no. 1-10-72-61-22), following local regulatory standards. Participants gave informed consent per the Helsinki Declaration (2013).

### Study population and inclusion criteria

A total of 302 consecutive males from couples undergoing IVF (176 Hewitt, 75 Skive, 51 Horsens) were included. Additionally, 126 healthy sperm donors with confirmed live births (Born and Cryos, Denmark) served as controls. Eligible participants were men aged 18–50 years and their female partners undergoing their first or only cycle of conventional IVF. Further inclusion criteria required that couples could provide informed consent and that men were able to produce freshly ejaculated sperm.

Exclusion criteria included the use of donor sperm, use of ICSI as the fertilization method, or split IVF/ICSI procedures, male age >50 years, female age >40 years, a history of repeated IVF failure, or semen parameters outside the ranges considered suitable for conventional IVF according to the protocols of the participating centres. Details of semen parameters for couples receiving conventional IVF across participating centres are summarized in the Supplementary Table S1. This table presents the available descriptive data, including minimum sperm concentration (million/ml) and total motility for participating couples, as recorded at each centre. These parameters were used to confirm eligibility for conventional IVF but were not included as predictors in the analytical models.

Male smoking history was collected using a questionnaire at enrolment; however, because complete smoking data were not available for all participants across centres and exploratory analyses did not show a significant association with SDF measures (Supplementary Tables S2, S3, and S4), smoking variables were not included in the primary predictive models.

### Outcome

The primary outcome was the probability of an live birth per initiated treatment cycle. This outcome was defined as the delivery of at least one live-born neonate (irrespective of gestational age), resulting from a single initiated IVF cycle.

### Semen sample collection and semen analysis

Semen samples were provided for the planned IVF. Specimens were collected following an ejaculatory abstinence interval of 1–3 days, in accordance with routine clinical practice at the participating IVF centres for treatment cycles. Although the WHO Laboratory Manual (6th edition) recommends an abstinence interval of 2–7 days for routine assessment of conventional semen parameters, it does not provide specific guidance on the duration of abstinence for SDF testing (WHO, 2021). Shorter abstinence intervals have been associated with lower levels of SDF and are increasingly adopted in IVF and ICSI settings to optimize sperm DNA integrity at the time of fertilization (Sørensen *et al*., 2023; Lo Giudice *et al*., 2024).

Semen analysis was performed within 1 h of ejaculation using Computer Aided Semen Analysis (CASA) using the Semen Analysis Machine Intelligence system (SAMi 1.0; Version 7; 2021; Hawksley and Mitrone Healthcare Ltd, UK) or the Sperm Class Analyzer (SCA) (Microptic S.L., Spain). Volume was assessed by weight using a UKAS-calibrated balance to 2 decimal places. pH was measured using MQuant^®^ (Merck, Germany) pH paper in the range between 6.0 and 10. Sperm concentration was assessed using a validated haemocytometer after appropriate dilution. Motility was observed on replicate slides in wet preparation. Morphology was assessed using Diff-Quik stain (RAL Diagnostics, France). Result thresholds were assigned against the 5th percentile: Semen Volume (1.4 ml), Sperm Concentration (16×10^6^ per ml), Total Sperm Number (39×10^6^ per ejaculate), Progressive Motility (30%), Morphology (4% Normal Forms).

Semen analyses were conducted in accordance with the principles outlined in the *Semen Analysis Methodology Checklist for Authors, Reviewers and Editors* (modified from Björndahl *et al.*, 2022). The procedures followed, as far as possible, the ISO Standard on basic semen examination and the WHO Laboratory Manual for the Examination and Processing of Human Semen, sixth edition (WHO, 2021). All three participating IVF centres used clinically validated CASA platforms run under routine internal quality control, and each centre operated within accredited laboratory quality-management frameworks. Because CASA parameters were not included in the predictive models, no inter-centre CASA comparison study was performed. The reference to WHO manual in this study pertains to laboratory methodology and quality standards for semen analysis rather than to a prescriptive abstinence interval for SDF assessment.

### Sperm DNA fragmentation testing

#### Freezing

After liquefaction, a 0.5–1.0 ml aliquot was transferred to pre-cooled cryovials and snap-frozen by direct immersion in liquid nitrogen within ≤60 min of collection. All samples from patients and healthy donors followed the same freeze–thaw standard operating procedures (SOPs). They were transported in dry shippers to the central laboratory. Neutral comet performance on frozen–thawed semen has been reported to be stable under standardized lysis/electrophoresis conditions (Ribas-Maynou *et al.*, 2014); the central laboratory’s monthly QC with pooled controls confirmed assay reproducibility across runs. Samples were transferred using anonymized identifiers only, and laboratory personnel were blinded to all clinical information, including fertility status, treatment allocation, and IVF outcomes. Assignment of dsSDF metrics (ACS and IOD) was completed before data linkage and outcome analysis.

#### Analysis

Samples were analysed in duplicate on masked slides using the Extend^®^ comet assay. Examen is ISO 13485:2016 certified for the design, development, and manufacture of *in vitro* diagnostic DNA-based assays for sperm cell analysis (Certificate No: MD 723205, issued by BSI Assurance UK), ensuring the highest standards in *in vitro* diagnostic test development. The Extend^®^ neutral comet assay was performed within Examen’s ISO 15189-accredited laboratory, an internationally recognized standard that verifies the quality, competence, and integrity of medical laboratories.

#### Measurement of double-stranded sperm DNA fragmentation

Neutral comet assays were performed using standardized Extend^®^ procedures. To establish efficient neutral comet assay experimental conditions, the procedures described by Ribas-Maynou *et al*. (2014) were applied with modifications in the initial lysis steps, and electrophoresis was optimized. Briefly, the semen sample concentration was adjusted to 2 × 10^6^/ml in PBS. Fully frosted slides (Leica Microsystems, UK) were layered with 150 μl of 1% normal melting agarose (NMA) (Sigma Aldrich, USA) and immediately covered with a coverslip. Once the NMA had solidified, the coverslip was removed and immediately layered with a mixture of 10 μl of diluted sample (2 × 10^6^/ml in PBS, Sigma Aldrich, USA) and 75 μl of 0.95% low-melting agarose (LMA) (Sigma Aldrich, USA). The slides were quickly covered with a coverslip and allowed to solidify at room temperature (RT). Once the LMA had solidified, the coverslip was removed, and the slide was incubated for 30 min at RT in lysis buffer I (0.8M Tris-HCl, 16.25 mM DTT, 1% SDS, pH 7.5), followed by 30 min at RT in lysis buffer II (0.4M Tris-HCl, 2M NaCl, 16.25M DTT, 50 mM Na_2_EDTA, pH 7.5), followed by 30 min at RT in lysis buffer III (0.4M Tris-HCl, 1% SDS, 0.8 mM DTT, pH 7.5) in the fume hood. Next, slides were washed in 0.9% NaCl solution three times for 10 min and then washed three times for 5 min in PBS. Following this, slides were rinsed in cold 1× TBE electrophoresis buffer (89 mM TRIS-HCl, 89 mM Boric acid, 2 mM Na_2_EDTA, pH 7.5) for 10 min. Electrophoresis was carried out with cold 1× TBE electrophoresis buffer. Slides were submerged, and electrophoresis was run at 31 V for 17 mins (1 V/cm), with the current adjusted to 40 mA. Following electrophoresis, slides were stained with 30 μl of 0.25× SYBR Gold stain (Thermo Fisher Scientific, USA). All steps were carried out in a temperature-controlled environment to prevent induction of DNA damage during processing.

Fifty comet images per slide were analysed in duplicate using KometGLP image analysis software (Andor Technology, Belfast, UK) by a masked, experienced user. During the analysis, only individual, non-superposed comets were included. Debris, superposed comets, or other artefacts were discarded from the counting process. Any issues noted with the gel standard, shape of the comet, or brightness of the comets resulted in the slides being discarded and the comet assay repeated to ensure high-quality results were derived for all research samples. The ACS (average %Tail DNA across cells) and IOD (% cells with %Tail DNA >10%) were computed for the 100 sperm cells analysed with KometGLP software, as previously described (Drakeley *et al.*, 2026). IOD is a metric used to indicate the proportion of spermatozoa from the patient sample that have a high degree of dsSDF. It reports the percentage of cells that display %TailDNA greater than 10% for 100 cells analysed, with this threshold previously defined (Drakeley *et al.*, 2026).

#### Image analysis

Comet images were analysed using KometGLP software, which uses photometric data to generate quantitative results where the user gates the desired comet, allowing KometGLP to identify the comet head and comet tail. Once identified, the intensity profile of the tail is analysed and is presented as the %Tail DNA. SDF analysis was performed at a central laboratory by trained personnel using the Extend^®^ neutral comet assay under predefined analytical protocols.

#### Technical feasibility

For the technical feasibility of the neutral comet assay, samples yielding fewer than 100 scorable sperms after standard slide preparation were classified as insufficient for dsSDF assessment and excluded from dsSDF analyses; all such exclusions were logged. No *a priori* inclusion or exclusion thresholds were applied for dsSDF testing concerning semen concentration, motility, or morphology beyond the feasibility rule above.

### Healthy donors

Healthy donor controls were sourced from Born sperm bank, Denmark. Eligibility required age 18–45 years, a sexual abstinence of 2–7 days, and absence of febrile illness within 3 months; donors with urogenital disease, varicocele grade ≥2, or current antioxidant/hormonal therapy were excluded. Semen collection, handling, and snap‑freezing followed the same SOPs as the IVF cohort; dsSDF assays were run under identical conditions with duplicate slides per sample. ACS (average %Tail DNA) and IOD (% cells with %Tail DNA > 10%) were calculated using KometGLP. This control group provided a benchmark for dsSDF distribution in proven fertile males under matched analytical and handling conditions.

### Statistical analysis

Normality was checked via the Shapiro–Wilk test. Non-parametric methods (Mann-Whitney U, Kruskal-Wallis, Dunn’s *post hoc*) were used to compare groups. To evaluate the predictive value of dsSDF on live birth, binary logistic regression was performed using live birth (yes/no) as the dependent variable. The primary independent variables were ACS and IOD measured by the Extend^®^ comet assay and expressed as either ACS (%) or IOD (%).

Models were adjusted for clinically relevant covariates, including female age and male age. Female age was modelled either as a continuous predictor or using clinically relevant categories: 18–34, 35–37, and 38–40, with the 18–34 group as the reference. These categories align with widely adopted reporting conventions in IVF and reflect clinically meaningful thresholds for prognosis and treatment decision-making. To confirm this approach, we compared the continuous predictor and categorical specification with an alternative model in which female age was entered as a restricted cubic spline with four knots (5th, 35th, 65th, 95th percentiles). Likelihood ratio testing showed that the spline did not significantly improve model fit, and discrimination/calibration indices were unchanged. Given the lack of added explanatory value and the greater clinical interpretability of age as a linear predictor or as categorical groups, we retained these specifications in all primary models.

Site-stratified models were also constructed to assess consistency across the three participating clinics (Skive, Horsens, Hewitt). Clinic effects were tested both as fixed indicators (with Hewitt as reference) and as a random intercept in mixed-effects logistic regression. The random-effect variance was estimated at or near zero, indicating minimal between-site heterogeneity.

Sensitivity analyses included modelling IOD as a continuous predictor and as a dichotomous variable using the pre-specified clinical threshold of 6%. Additional thresholds between 4% and 10% were also explored to assess robustness. Potential interactions between SDF and male or female age were also tested. As additional sensitivity analyses, sperm concentration and total motility were included individually in the multivariable logistic regression models to assess whether conventional semen parameters influenced the association between dsSDF and live birth.

Model results are presented as odds ratios (ORs) with 95% CIs. Discrimination was assessed using the area under the receiver operating characteristic curve (AUC), with DeLong’s method comparing AUCs to determine performance improvement. Calibration was evaluated using bootstrap resampling. Predicted probabilities are shown only within the observed data range, and uncertainty at the margins is explicitly conveyed through CIs to avoid over-interpretation where data density is limited. Analyses were conducted in R (v4.3.0). Analysis of variance and two-sided *P* < 0.05 was considered statistically significant.

## Results

### Patient characteristics

Across the three participating clinics, notable demographic and biological differences were observed among the IVF cohorts. Female age was highest at the Hewitt Fertility Centre, with a median of 35 years (IQR: 32–38), compared to Horsens (31; IQR: 29–35; *P* < 0.001) and Skive (31; IQR: 28–35; *P* < 0.001). When stratified by age group, Skive and Horsens had a higher proportion of younger patients: 71% and 69% of women were aged 18–34 years, respectively, compared with only 48.3% at Hewitt (Table 1). A similar pattern was seen in male age, with the Hewitt cohort being older (36; IQR: 33–40) than Horsens and Skive (32 years; IQR: 30–36 and 29–35, respectively; *P* < 0.001) (Table 1). The median age of the fertile donor group was 24 years (interquartile range [IQR]: 22–28), consistent with their role as a reference population of healthy, fertile men (Table 1).

**Table 1.** Patient characteristics across clinics and live birth outcomes.

Characteristic	Hewitt (n = 176)	Horsens (n = 51)	Skive (n = 75)	*P*-value (clinic)	Overall; IVF patients (n = 302)	Fertile donors (n = 125)	*P*-value (IVF patients vs donors)	No live birth (n = 211)	Live birth (n = 91)	*P*-value (outcome)
Female age (years)	35 (32–38)	31 (29–35)	31 (28–35)	<0.001	34 (30–37)	–	–	34 (31–38)	33 (28–35)	<0.001
Male age (years)	36 (33–40)	32 (30–36)	32 (29–35)	<0.001	35 (31–38)	24 (22–28)	<0.001	35 (31–39)	34 (31–37)	0.20
ACS (%)	6.03 (5.05–7.24)	5.70 (4.90–6.30)	6.20 (5.00–7.70)	0.2	6.00 (5.00–7.10)	3.42 (3.03, 3.78)	<0.001	6.12 (5.11–7.27)	5.70 (4.90–6.70)	0.042
IOD (%)	9 (6–14)	8 (6–11)	9 (5–15)	0.3	9 (6–13)	1 (0–2)	<0.001	9 (6–14)	8 (5–12)	0.076
Live birth, n (%)	45 (26%)	17 (33%)	29 (39%)	0.10	91 (30%)	–	–	–	–	–
Female age group 18–29, n (%)	17 (9.7%)	16 (31%)	32 (43%)	–	65 (22%)	–	–	36 (17%)	29 (32%)	<0.001
Female age group 30–34, n (%)	68 (39%)	19 (37%)	21 (28%)	–	108 (36%)	–	–	73 (35%)	35 (38%)	–
Female age group 35–37, n (%)	47 (27%)	9 (18%)	11 (15%)	–	67 (22%)	–	–	47 (22%)	20 (22%)	–
Female age group 38–40, n (%)	44 (25%)	7 (14%)	11 (15%)	–	62 (21%)	–	–	55 (26%)	7 (8%)	–

Values expressed as median (Q1, Q3) or n (%). ACS, Average Comet Score; IOD, Incidence of Damage. Clinic-level comparisons used Kruskal–Wallis rank-sum or Fisher’s exact test. Outcome-level comparisons used Wilcoxon rank-sum, Fisher’s exact, or chi-squared test as appropriate.

The dsSDF levels, as measured by the Extend^®^ neutral comet assay, varied modestly across sites. The median ACS was highest in Skive (6.20%; IQR: 5.00–7.70), followed closely by Hewitt (6.03%; IQR: 5.05–7.24), with Horsens showing the lowest median ACS (5.70%; IQR: 4.90–6.30). IOD showed similar trends: 9.0% (IQR: 6.0–14.0) in Hewitt, 9.0% (IQR: 5.0–15.0) in Skive, and slightly lower at 8.0% (IQR: 6.0–11.0) in Horsens. This can be contrasted with the healthy fertile donors (n = 125), where the median ACS and IOD were 3.42% (IQR: 3.03–3.78%) and 1% (IQR: 0–2%), respectively, which was significantly lower than all three fertility clinics (all *P* < 0.001 by Dunn’s *post hoc* testing).

### Impact of dsSDF on live birth

Among the 302 couples included, 91 (30%) achieved an live birth. The median Extend^®^ ACS was lower among couples who achieved an live birth (5.70% vs 6.12%; *P* = 0.042), whereas IOD did not differ significantly (8% vs 9%; *P* = 0.076) (Table 1). In the adjusted logistic regression analysis, both continuous measures of dsSDF, using ACS and IOD, were independent predictors of IVF success. In the ACS model, each 1% increase in ACS was associated with a 16% reduction in the odds of live birth (OR 0.84, 95% CI 0.72–0.97, *P* = 0.026), which was statistically significant (Table 2). Similarly, in the IOD model, each 1% increase in IOD was associated with a 5% reduction in the odds of live birth (OR 0.95, 95% CI 0.90–0.99, *P* = 0.025), which was statistically significant (Table 2). Inclusion of sperm concentration or total motility in the multivariable models did not materially change the association between dsSDF and live birth, and effect estimates remained unchanged (Supplementary Tables S5 and S6).

**Table 2.** Odds ratios for live birth according to Extend^®^ neutral comet assay metrics (ACS and IOD), adjusted for parental age and clinic.

Characteristic	ACS model; OR (95% CI)	*P*-value	IOD model; OR (95% CI)	*P*-value
Extend ACS (%)	0.84 (0.72–0.97)	0.026	–	–
Extend IOD (%)	–	–	0.95 (0.90–0.99)	0.025
Female age (years)	0.86 (0.78–0.94)	<0.001	0.86 (0.78–0.94)	<0.001
Male age (years)	1.08 (1.01–1.16)	0.033	1.08 (1.01–1.17)	0.029
Clinic (ref: Hewitt):				
Horsens	1.09 (0.52–2.20)	0.8	1.09 (0.52–2.20)	0.8
Skive	1.58 (0.83–2.99)	0.2	1.56 (0.82–2.94)	0.2

OR, odds ratio; ACS, Average Comet Score; IOD, Incidence of Damage. Models adjusted for female and male age and clinic site (reference: Hewitt).

**Figure 1.**
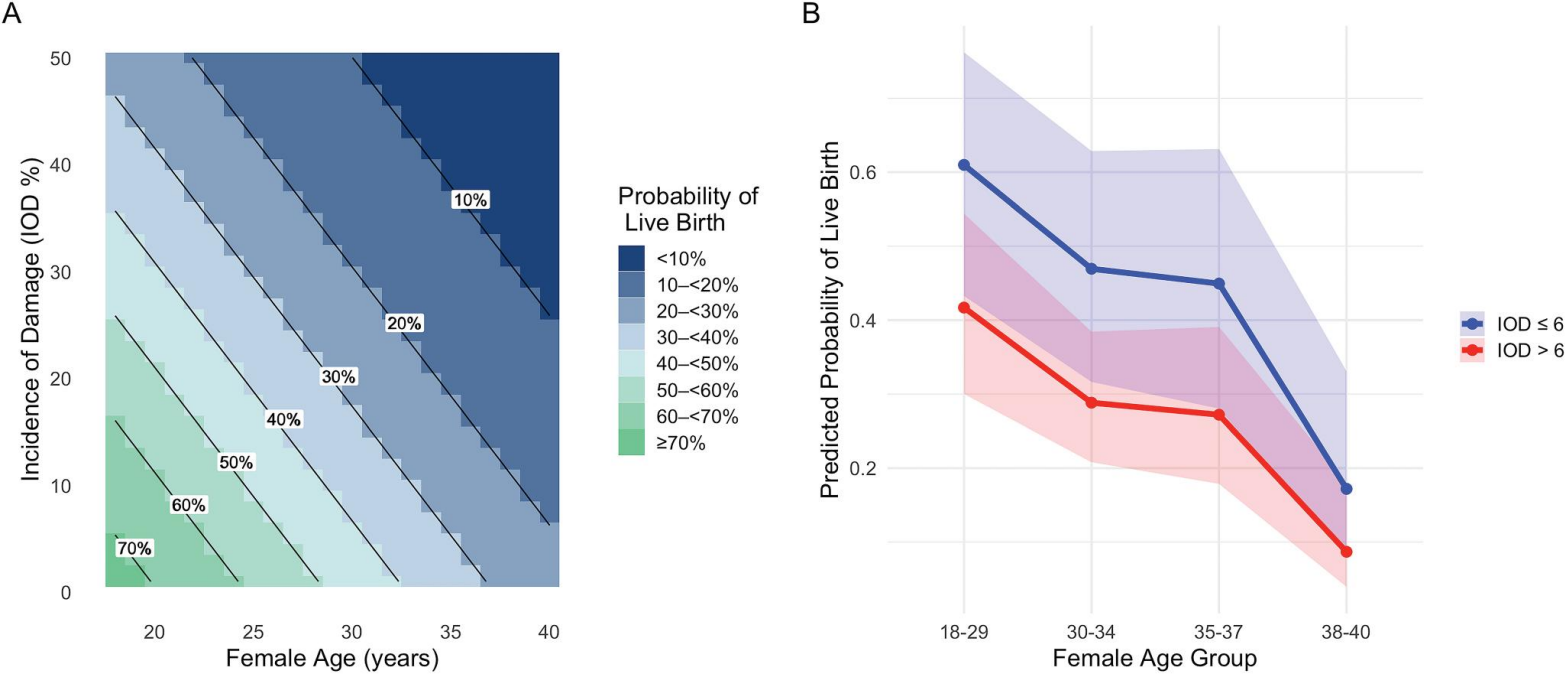
**Predicted probability of live birth according to female age and sperm double-stranded DNA fragmentation (dsSDF)**. (**A**) Colour-coded heat map illustrating the predicted probability of live birth according to female age and sperm double-stranded DNA fragmentation, assessed using the Incidence of Damage (IOD) scores. The labelled probability contour lines (isolines) were derived directly from the fitted multivariable logistic regression model based on Extend^®^ neutral comet assay data from three fertility centres. (**B**) Predicted probability of live birth following IVF, stratified by female age group and IOD category. Predictions were generated from logistic regression models using categorical female age bands (18–29, 30–34, 35–37, and 38–40 years) and IOD thresholds (≤6% vs >6%). Solid lines represent model-predicted probabilities, while shaded bands indicate the 95% CIs around these estimates.

Although the multivariable models indicated that both Extend^®^ ACS and IOD were independently associated with live birth after adjustment for female age, these estimates are driven primarily by the central part of the age distribution. As shown in Table 1, most women were between 30 and 37 years of age, with fewer patients in the youngest (18–29 years) and oldest (38–40 years) groups. Consequently, predictions at the extremes of age and DNA fragmentation are supported by sparser data and should be interpreted with caution. The widening CIs around these predictions (Fig. 1) reflect this uncertainty, and we therefore consider the overall shape and direction of the associations to be more robust than the precise magnitude of effect at the margins.

### Impact of parental age on live birth

Women from couples who achieved an live birth were significantly younger (median 33 years, IQR 28–35, Table 1) compared to those without an live birth (median 34.0 years, IQR 31–38, *P* < 0.001). When stratified by age group, women aged 18–34 years accounted for 70% of those achieving an live birth. Female age was consistently associated with the probability of achieving an live birth, with both ACS and IOD as covariates. A 1-year increase in age was associated with a 14% reduction in odds of achieving an live birth, irrespective of the ACS or IOD metric used, which was statistically significant (OR 0.86, 95% CI 0.78–0.94, *P* < 0.001) (Table 2).

Male partners were slightly younger in the live birth group (34.0 vs 35.0 years; *P* = 0.2) (Table 1). It should be noted, however, that male and female ages correlated highly by Spearman’s Rho analysis. Therefore, the impact of male age alone on live birth outcomes is difficult to ascertain from this study, as the two factors are intrinsically linked. Male age showed a weak positive trend with ACS as a covariate (OR 1.08, 95% CI 1.01–1.16, *P* = 0.033) and with IOD as a covariate (OR 1.08, 95% CI 1.01–1.17, *P* = 0.029), both of which were statistically significant (Table 2).

### Impact of clinic on live birth

The live birth rates recorded from the cohort of 302 couples differed at the three clinic sites, but this was not statistically significant (*P* = 0.10). A higher proportion of live births was reported at Skive (39%), followed by Horsens (33%) and Hewitt (26%), when considering the number of live births as a proportion of patients at each clinic (Table 1).

The impact of the clinic on ORs for live birth, with ACS and IOD as covariates, was also considered, using the Hewitt site as a reference for comparison. The clinic-level effects were not statistically significant; Horsens did not differ from Hewitt, while Skive also showed no significant difference in live birth odds with ACS as covariate (OR 1.58, 95% CI 0.83–2.99, *P*=0.2) and IOD as covariate (OR 1.56, 95% CI 0.82–2.94, *P*=0.2) (Table 2). Sensitivity analyses using mixed-effects models with clinic as a random intercept confirmed negligible between-centre heterogeneity.

### Impact of female age in combination with SDF on live birth

The impact of female age and male-derived Extend^®^ IOD on the respective probability of live birth following IVF is depicted in the decision matrix in Fig. 1A. This could be utilized as a tool to show the respective probability of live birth, aiding in decision-making regarding possible treatment pathways.

As an example, this decision matrix shows that a woman aged 25 years, whose male partner has an IOD of 5%, will have a probability of an live birth between 60% and 70%. However, if the 25-year-old woman’s partner has an IOD of 20%, the probability of an live birth is reduced to between 30% and <40%. For a 35-year-old woman with a male partner with an IOD of 5%, the probability of live birth is 30-<40%. However, if the IOD is increased to 20%, this reduces the probability of live birth to 10-<20%. This highlights the involvement of dsSDF, as shown by IOD, along with female age, in the predicted probability of live birth outcomes following IVF (Fig. 1A).

For clinical interpretability, a simplified model using dichotomized IOD (≥6% vs <6%, based on our previous cut-off point) (Drakeley *et al*., 2026) was also examined. In this specification, men with IOD ≥6% had less than half the odds of live birth compared to those below the threshold both when female age was used as a continuous variable (OR 0.51, 95% CI 0.28–0.94, *P* = 0.029; Table 3), or when used as a categorical variable (OR 0.46, 95% CI 0.24–0.86, *P* = 0.015; Table 3).

**Table 3.** Odds ratios for live birth with dichotomized Extend^®^ neutral comet assay IOD (≥6%), adjusted for female age (continuous or categorical).

Characteristic	Continuous age model; OR (95% CI)	*P*-value	Categorical age model; OR (95% CI)	*P*-value
Extend^®^ IOD <6%	–	–	–	–
Extend^®^ IOD ≥6%	0.51 (0.28–0.94)	0.029	0.46 (0.24–0.86)	0.015
Female Age (per year)	0.90 (0.84–0.95)	<0.001	–	–
Female Age Group (ref: 18–29)	–	–	–	–
30–34	–	–	0.57 (0.30–1.08)	0.085
35–37	–	–	0.52 (0.25–1.07)	0.080
38–40	–	–	0.13 (0.05–0.33)	<0.001

OR, odds ratio; ACS, Average Comet Score; IOD, Incidence of Damage. Models adjusted for female age as either continuous (per year) or categorical groups (reference: 18–29 years).

When examining categorical female age groups with dichotomized Extend^®^ IOD and comparing to women aged 18–29 years, the odds of live birth were non-significantly lower for those aged 30–34 years (OR 0.57, 95% CI 0.3–1.08, *P* = 0.085) and 35–37 (OR 0.52, 95% CI 0.25–1.07, *P* = 0.08), but markedly reduced for women aged 38–40 years (OR 0.13, 95% CI 0.05–0.33, *P* < 0.001) (Table 3).

Figure 1B illustrates the interaction between each female age group and the dichotomized IOD score. Within each group, patients with IOD ≤ 6% (blue) show higher predicted probabilities compared to those with IOD > 6% (red), highlighting the independent predictive value of dsSDF. Notably, the sharpest decline in success is seen after age 37, particularly when combined with elevated dsSDF. This pattern supports the utility of incorporating both continuous and stratified age measures when modelling IVF outcomes.

## Discussion

### Main findings

In this multicentre prospective cohort, higher dsSDF, as measured by the neutral comet assay, was associated with lower odds of live birth after IVF. Both the mean damage burden, as indicated by ACS, and the distributional burden, as indicated by IOD, showed independent associations after adjustment for parental age and centre. Risk stratification using an IOD cut-off of 6% identified a group with a substantially reduced likelihood of live birth. The adverse association appeared to intensify with advancing female age, which is biologically plausible given the age-related decline in oocyte DNA-repair capacity. Together, these results support dsSDF as a clinically relevant biomarker that complements conventional semen parameters.

The 6% cut-off was identified in our previous work as a clinically meaningful threshold (Drakeley *et al*., 2026); however, it has not yet undergone external validation and should therefore be interpreted as provisional. In the present study, the cut-off is used primarily to dichotomize the population for visualization and to illustrate the potential clinical utility of stratification. As with many continuous biomarkers, any threshold represents an operational tool rather than a biological boundary: dsSDF exists on a continuum, and increasing values reflect progressively greater sperm DNA damage and therefore greater clinical concern.

### Context with prior evidence

Comparative evidence across assays specifically targeting dsSDF remains limited and heterogeneous. Studies using differential comet assay conditions have provided important biological context, with Casanova *et al*. (2019) demonstrating that dsSDF but not single-stranded DNA damage was associated with impaired embryo morphokinetics and reduced implantation in ICSI cycles, although live birth was not assessed as a primary endpoint. Neutral comet studies have further linked elevated dsSDF to RPL, rather than to IVF live-birth outcomes (Drakeley *et al*., 2026). More recently, emerging commercial platforms designed to preferentially quantify dsDNA damage, such as semi-automated quantification of double-stranded break load using DNA-release kinetics, have reported associations with embryo aneuploidy and increased risk of miscarriage or failure to achieve ongoing pregnancy (Wang *et al*., 2023; Lee *et al*., 2025). However, these studies have generally been limited by sample size, and none have evaluated live birth as the primary outcome in conventional IVF.

In this context, the present study extends the existing literature by providing multicentre, prospective evidence linking dsSDF measured under neutral comet conditions to live birth after conventional IVF, while acknowledging that comparative validation across dsDNA assays remains an evolving area requiring further independent studies. Whereas mean-based indices (e.g. ACS) summarize central tendency, IOD captures a distributional property: the proportion of sperm exceeding a biologically meaningful damage threshold (Drakeley *et al.*, 2026). Because fertilization and embryo formation are single‑cell events, a tail of highly damaged sperm may exert an outsized clinical impact even when the average is modest. In our workflow, IOD quantified the percentage of sperm cells measured by the KometGLP software, where the %Tail DNA was 10%. In adjusted models, each increase in IOD was associated with lower odds of live birth, and men with an IOD ≥6% had substantially lower odds compared to those with an IOD <6%.

Clinically, IOD offers two advantages: (i) it translates into actionable counselling (‘how many spermatozoa are above a damage line’), aligning with the single‑cell nature of ART; and (ii) it is more robust to skew than mean‑based metrics, mitigating scenarios where a minority of severely damaged sperm inflate risk. The interaction with female age is biologically plausible: as oocyte DNA‑repair capacity declines, a higher incidence of severely damaged sperm may exceed repair reserves, compounding risk. While the IOD ≥6% threshold is useful for stratification, we recommend external calibration and reporting of continuous IOD to preserve transportability.

### Biological interpretation

Most commonly used SDF assays report a global measure of DNA damage that reflects a composite of single- and double-strand breaks (Esteves *et al*., 2021). In contrast, the neutral comet assay used in this study preferentially detects dsSDF, thereby providing discriminatory biological information by isolating a specific subtype of DNA damage rather than total strand break burden. This discriminatory capability is biologically relevant, as dsSDF are less readily repaired after fertilization and have been implicated in chromosomal instability and adverse reproductive outcomes, whereas single-strand lesions may be more amenable to oocyte-mediated repair (Aitken and De Iuliis, 2010).

Following fertilization, the oocyte’s repair capacity declines with maternal age (Evenson *et al*., 2002; Aitken and De Iuliis, 2007). An increased incidence of severely damaged sperm may therefore exceed repair reserves in older oocytes, amplifying the impact of dsSDF on embryo competence.

In this study, male age showed a smaller effect than female age in our models; this decouples the impact of chronological ageing from that of molecular damage, underscoring the potential for therapeutic intervention and diagnostic refinement. Future work could examine whether dsSDF partially mediates any male-age effect through formal mediation analyses. In a previous single-site study, we demonstrated that dsSDF was not significantly associated with male age (Drakeley *et al.*, 2026). This was corroborated here with a low correlation between male age and the dsSDF covariates observed (Spearman’s Rho = 0.17). As there is a tendency for partners to be in a similar age bracket, this results in male and female ages being highly correlated. Therefore, a caveat of the logistic regression analysis is that this correlation has the potential to impact the estimation of the impact of male age.

Beyond oocyte-mediated repair capacity, emerging molecular and embryological data provide additional insight into how elevated dsSDF may compromise embryo competence and chromosomal stability. Time-lapse studies have reported that embryos derived from sperm with higher SDF exhibit altered cleavage dynamics, which is increasingly recognized as a marker of compromised embryo viability (Chamayou *et al*., 2023). At the chromosomal level, paternal DNA integrity has been implicated in early mitotic fidelity, with experimental and clinical data indicating that mis-repaired double-strand breaks in the paternal genome can lead to chromosomal instability during the first embryonic divisions (Middelkamp *et al*., 2020). This instability may manifest as mitotic aneuploidy or mosaicism rather than meiotic errors, providing a plausible mechanistic link between dsSDF, impaired embryo development, and reduced implantation potential (Baran *et al*., 2025). While preimplantation genetic testing for aneuploidy (PGT-A) primarily captures chromosomal copy-number abnormalities at the blastocyst stage and cannot directly attribute causality to paternal DNA damage, several studies have reported associations between increased SDF and higher rates of embryo aneuploidy (Wan *et al*., 2025). Together, these observations support the concept that dsSDF may exert its detrimental effects by perturbing early embryonic cell-cycle regulation and chromosomal stability, thereby compromising developmental competence and live birth potential.

It is important to note that the available evidence comparing dsSDF with global SDF measures is limited, and differences in predictive performance may reflect variation in assay methodology, clinical endpoints, and underlying biological mechanisms (Robinson *et al*., 2012; Esteves *et al*., 2021). Our findings therefore support dsSDF as a complementary marker that adds biological resolution, rather than as a universally superior measure of sperm DNA damage.

### Clinical implications

We propose a pragmatic use-case for dsSDF testing in IVF pathways, which integrates female age. For couples with low IOD and younger female age, the prognosis is favourable, and conventional IVF can proceed with reassurance. Where IOD is elevated, particularly with older female age partners, clinicians can offer more realistic counselling and consider strategies to address potentially modifiable contributors (e.g. treatment of a clinical varicocele, management of genital tract infection, optimization of abstinence intervals, and lifestyle/exposures), while recognizing that high-quality evidence for specific interventions remains limited (Esteves *et al*., 2021). The use of ICSI as the fertilization method and selection technologies like microfluidics that reduce laboratory-measured dsSDF may also offer biological plausibility (Esteves *et al*., 2021; Nielsen *et al.*, 2023; Gisbert Iranzo *et al*., 2025) but should not be assumed to improve clinical outcomes without prospective trials (Pujol *et al*., 2022). The current data support the diagnostic/prognostic role of dsSDF rather than endorsing a particular intervention.

The decision matrix presented in Fig. 1a integrates two of the strongest determinants of IVF success, female age and dsSDF, measured as IOD, to provide an intuitive guide for clinicians and patients. By modelling predicted live birth across the full spectrum of female age and IOD values, the matrix highlights the compounding impact of these factors. While younger women with low IOD have the highest predicted probabilities of success (>40% probability of live birth), outcomes decline sharply with advancing maternal age or with elevated dsSDF, with the lowest probabilities seen when both risk factors are present.

From a clinical perspective, the matrix offers a framework for stratifying prognosis at the outset of treatment. Couples with favourable profiles (younger age and low IOD) may be reassured that they have a higher likelihood of success with standard IVF approaches. Conversely, couples in higher-risk categories can be counselled regarding their reduced probability of live birth and the potential value of targeted interventions or switching to ICSI. Ultimately, the decision matrix is not intended as a deterministic predictor for individual couples, but as a visual and data-driven aid to support personalized counselling, expectation management, and shared decision-making in the fertility clinic.

### Strengths and limitations

Strengths of this study include the prospective design, multicentre recruitment across three European clinics with differing demographic profiles, masked, duplicate neutral comet assessments within an ISO-accredited laboratory, and the use of live birth as the primary endpoint. We analysed dsSDF as both continuous (ACS, IOD) and categorical (IOD ≥ 6%) variables and examined the joint relationship with female age, producing a clinically usable probability matrix. Notably, conservative modelling approaches that balance clinical interpretability with statistical robustness. Live birth was modelled as a probabilistic outcome influenced by multiple biological determinants rather than as a deterministic binary state. Accordingly, inference was based on adjusted effect estimates and predicted probabilities derived from multivariable logistic regression, rather than on single-threshold discrimination metrics or individual-level prediction. Model-based visualizations were restricted to the observed data range to avoid extrapolation, and uncertainty was explicitly conveyed using CIs. Female age was examined using both continuous and flexible spline specifications, but simpler representations were retained where they offered comparable fit and greater clinical interpretability. Together, these choices were intended to support counselling and shared decision-making while avoiding over-precision or implied individual-level prediction. Notably, sensitivity analyses that included sperm concentration and total motility in the multivariable model did not materially change the dsSDF association, and effect estimates remained unchanged (Supplementary Tables S5 and S6).

Our study has several limitations. First, we did not collect or adjust for cycle-level covariates that influence live birth (e.g. IVF vs ICSI, number of oocytes retrieved, embryo transfer strategy, use of PGT-A, stimulation protocol), so residual confounding is possible.

Second, our analysis relates to the distribution of patients across age strata and DNA fragmentation levels. Although the overall IVF cohort was relatively large (n = 302), the number of women at the extremes of the age range was modest, and high dsSDF levels were uncommon among younger women. As a result, model-based predictions for combinations of advanced female age and high dsSDF are based on fewer observations and are correspondingly less precise. In addition, dsSDF and female age are expressed on different scales (continuous ACS/IOD values vs years), making direct comparison of their apparent effect sizes potentially misleading. For these reasons, we deliberately avoid over-interpreting the relative ‘strength’ of dsSDF versus female age and instead emphasize the consistency and direction of the dsSDF association across models. Larger, independent cohorts with broader age representation will be required to better define the relative contribution of paternal DNA integrity and maternal age to IVF prognosis.

Another limitation of this study is that it exclusively included couples undergoing conventional IVF, and therefore, the applicability of these findings to ICSI cycles requires cautious interpretation. While sperm selection may attenuate the impact of global DNA damage in ICSI cycles, it is primarily based on motility and morphology and does not directly assess DNA integrity. Consequently, spermatozoa harbouring dsSDF may still be selected for injection. Advanced sperm selection techniques, such as microfluidic sorting or other approaches designed to enrich for sperm with lower DNA fragmentation, may reduce laboratory-measured dsSDF; however, robust evidence demonstrating that these strategies translate into improved live birth outcomes remains limited (Gisbert Iranzo *et al*., 2025). Moreover, dsSDF reflects a distributional property of the ejaculate, and when the proportion of highly damaged sperm is increased, the probability of selecting an affected spermatozoon may remain non-negligible even with ICSI. Prospective studies designed explicitly for ICSI populations, including those incorporating advanced sperm selection methods, are needed to determine whether and to what extent such approaches modify the prognostic value of dsSDF.

Methodological considerations relate to the ejaculatory abstinence interval and use of CASA systems. In this study, the IVF cohort provided semen samples after 1–3 days of abstinence, reflecting contemporary IVF practice aimed at minimizing SDF, whereas fertile donor controls followed a 2- to 7-day abstinence interval in accordance with standard sperm bank protocols and WHO recommendations for donor screening. As longer abstinence intervals are consistently associated with higher levels of SDF (Sørensen *et al.*, 2023; Lo Giudice *et al.*, 2024), this difference would be expected to increase SDF levels in donors and thus bias comparisons towards the null. Notably, despite the longer abstinence interval, fertile donors exhibited substantially lower dsSDF levels than the IVF cohort, supporting the robustness of the observed associations. Nonetheless, differences in abstinence duration should be considered when interpreting absolute SDF values across populations. Although CASA systems were used for routine semen assessment rather than manual semen analysis, as recommended by the WHO (WHO, 2021) as the reference method, these parameters were reported descriptively only and did not contribute to the inferential analyses, limiting their impact on the study’s primary findings.

Lastly, although smoking history was collected prospectively, incomplete data availability across centres and the low prevalence of current smokers precluded inclusion of smoking variables in the primary models. Notably, although smoking has been associated with impaired semen quality and SDF in other studies (Sharma *et al.*, 2016; Szabó *et al.*, 2023), exploratory analyses in the present cohort did not demonstrate a significant relationship with dsSDF measures (Supplementary Tables S2, S3, and S4), likely reflecting the low prevalence of current smokers and incomplete data availability.

Future research should focus on external calibration/validation of IOD thresholds across laboratories, inter-laboratory ring-trials for assay harmonization, and prospective studies testing management algorithms informed by dsSDF (including age-integrated counselling). When feasible, inclusion of embryo developmental metrics and aneuploidy endpoints may clarify mechanistic pathways linking dsSDF to live birth outcomes.

## Conclusion

In this multicentre prospective IVF cohort, higher dsSDF measured by the neutral comet assay was independently associated with lower odds of live birth after adjustment for parental age and centre. Both ACS and IOD conveyed risk, with IOD offering a pragmatic distribution-based stratifier that aligned with the single-cell nature of fertilization. The age-dependent pattern we observed, with stronger adverse associations at older female ages, is biologically plausible given the progressive decline in oocyte DNA-repair capacity. This supports integrating male genomic integrity with female age when counselling couples. Our findings position dsSDF as a clinically relevant complement to conventional semen parameters in IVF pathways, particularly for prognosis and shared decision-making. Nevertheless, the observational design precludes causal inference, and the IOD cut-point used for risk stratification requires explicit pre-specification and external calibration before broad adoption. Harmonization of assay procedures, inter-laboratory quality assurance, and prospective evaluations of care pathways informed by dsSDF, ideally including embryo development and aneuploidy endpoints, are warranted. Pending such work, we recommend reporting both continuous ACS and IOD, modelling female age flexibly, and using dsSDF results to support counselling.

## Supplementary Material

deag046_Supplementary_Table_S1

deag046_Supplementary_Table_S2

deag046_Supplementary_Table_S3

deag046_Supplementary_Table_S4

deag046_Supplementary_Table_S5

deag046_Supplementary_Table_S6

## Data Availability

Due to legal restrictions under the Danish Data Protection Act and the EU General Data Protection Regulation (GDPR), the individual-level clinical datasets used in this study cannot be shared publicly, as they contain potentially identifiable patient information. Access to the data requires prior approval from the Danish regional ethics committees and the institutional data controllers at the participating fertility centres. Aggregated or anonymized data underlying the analyses presented in this article may be made available by the corresponding author upon reasonable request and with permission from the relevant data authorities.
