# Supplementary material for: Double-stranded sperm DNA fragmentation measured with neutral comet assay as a predictor of IVF outcomes: evidence from three European clinics in a multi-centred prospective study
Source: Hum Reprod. 2026 Mar 28;41(5):677–88. doi: 10.1093/humrep/deag046 (PMC13139651; doi:10.1093/humrep/deag046)
Supplement: deag046_Supplementary_Table_S1 [file deag046_supplementary_table_s1.pdf]

Human Reproduction, 2026, 1–1
https://doi.org/10.1093/humrep/deag046
Supplementary Data

Supplementary Table S1. Semen characteristics of couples undergoing conventional IVF across participating centres.

Characteristic	Overall N = 302	Hewitt N = 176	Horsens N = 51	Skive N = 75	P-value[1]
**Abstinence interval (range; days)**	1–3	1–3	1–3	1–3	N/A
**Volume (ml)**					0.2
Median (Q1–Q3)	2.40 (1.80–3.00)	N/A	2.20 (1.70–2.80)	2.40 (1.80–3.10)	
Min, Max	0.40, 6.00	N/A	0.60, 6.00	0.40, 5.60	
**Sperm concentration (million/ml)**					<0.001
Median (Q1–Q3)	45 (28–70)	55 (34–81)	39 (25–60)	32 (18–50)	
Min, Max	4, 217	4, 217	9, 145	5, 121	
**Total motility (%)**					<0.001
Median (Q1–Q3)	65 (55–75)	63 (55–72)	62 (50–71)	78 (63–87)	
Min, Max	24, 98	24, 90	24, 83	28, 98	
**Progressive motility (%)**					
Median (Q1–Q3)	53 (45–62)	53 (45–62)	N/A	N/A	
Min, Max	18, 85	18, 85	N/A	N/A	
**Total motile sperm count (TMSC, million)**					0.3
Median (Q1–Q3)	49 (30–84)	N/A	48 (30–70)	52 (29–102)	
Min, Max	2, 250	N/A	12, 220	2, 250	
**Post-wash concentration (million/ml)**					0.056
Median (Q1–Q3)	10 (6–20)	N/A	14 (8–20)	8 (6–16)	
Min, Max	2, 70	N/A	2, 70	3, 57	
**Post-wash TMSC (million)**					0.016
Median (Q1–Q3)	8 (4–19)	N/A	12 (6–20)	7 (3–16)	
Min, Max	1, 70	N/A	1, 70	1, 56	

Values represent descriptive semen characteristics of couples who underwent conventional IVF at each participating centre. Semen parameters were used to confirm clinical suitability for conventional IVF according to centre-specific protocols and were not included as predictors in the statistical models. Data availability varied across centres; only parameters routinely recorded and available are shown.

[1] Kruskal-Wallis rank sum test. Conventional semen parameters were measured using centre-specific CASA platforms under routine internal quality control; N/A, not available.
