# Supplementary material for: Double-stranded sperm DNA fragmentation measured with neutral comet assay as a predictor of IVF outcomes: evidence from three European clinics in a multi-centred prospective study
Source: Hum Reprod. 2026 Mar 28;41(5):677–88. doi: 10.1093/humrep/deag046 (PMC13139651; doi:10.1093/humrep/deag046)
Supplement: deag046_Supplementary_Table_S2 [file deag046_supplementary_table_s2.pdf]

OXFORD
human reproduction
Human Reproduction, 2026, 1–1
https://doi.org/10.1093/humrep/deag046
Supplementary Data

Supplementary Table S2. Live-birth outcomes according to smoking status and smoking cessation history.

Smoking status/ cessation history	Total n (%)	Live birth n (%)	No live birth n (%)	P-value*
Smoking status	126 (100.0)			0.10
Never smoked	77 (61)	48 (61.0)	29 (62.0)	
Stopped smoking	45 (36)	30 (38.0)	15 (32.0)	
Smoke daily	3 (2.4)	0 (0.0)	3 (6.4)	
Smoke e-cigarettes	1 (0.8)	1 (1.3)	0 (0.0)	
Cessation history	44 (100.0)			0.06
Cessation < 3 months	5 (11.0)	4 (13.0)	1 (7.1)	
Cessation 3–6 months	9 (20.0)	9 (30.0)	0 (0.0)	
Cessation 6–12 months	3 (6.8)	2 (6.7)	1 (7.1)	
Cessation > 1 year	27 (61.0)	15 (50.0)	12 (86.0)	

* P-values derived from chi-square or Fisher's exact test, as appropriate.
Smoking history was self-reported. Analyses were restricted to participants with available questionnaire data (n = 126). The number of current smokers was low, limiting statistical power.
