# Supplementary material for: Double-stranded sperm DNA fragmentation measured with neutral comet assay as a predictor of IVF outcomes: evidence from three European clinics in a multi-centred prospective study
Source: Hum Reprod. 2026 Mar 28;41(5):677–88. doi: 10.1093/humrep/deag046 (PMC13139651; doi:10.1093/humrep/deag046)
Supplement: deag046_Supplementary_Table_S3 [file deag046_supplementary_table_s3.pdf]

human reproduction

OXFORD

Human Reproduction, 2026, 1–1
https://doi.org/10.1093/humrep/deag046
Supplementary Data

Supplementary Table S3. Sperm DNA fragmentation parameters according to smoking status.

Smoking status	n (%)	ACS median (IQR)	IOD % median (IQR)
Never smoked	77 (61.1)	5.90 (4.80–6.80)	9.00 (6.00–12.00)
Smoke daily	3 (2.4)	5.70 (5.10–5.75)	7.00 (5.50–8.00)
Smoke e-cigarettes	1 (0.8)	4.90 (4.90–4.90)	8.00 (8.00–8.00)
Stopped smoking	45 (35.7)	6.10 (5.30–7.70)	9.00 (6.00–13.00)
P-value†	126 (100.0)	0.365	0.645

† *P*-values derived from Kruskal–Wallis test.
ACS, Average Comet Score; IOD, Incidence of Damage.
Smoking history was self-reported. No statistically significant differences in SDF parameters were observed across smoking categories.
