# Supplementary material for: Double-stranded sperm DNA fragmentation measured with neutral comet assay as a predictor of IVF outcomes: evidence from three European clinics in a multi-centred prospective study
Source: Hum Reprod. 2026 Mar 28;41(5):677–88. doi: 10.1093/humrep/deag046 (PMC13139651; doi:10.1093/humrep/deag046)
Supplement: deag046_Supplementary_Table_S4 [file deag046_supplementary_table_s4.pdf]

Human Reproduction, 2026, 1–1
https://doi.org/10.1093/humrep/deag046
Supplementary Data

Supplementary Table S4. Sperm DNA fragmentation parameters according to smoking cessation status.

Smoking cessation status	n (%)	ACS median (IQR)	IOD % median (IQR)
Stopped < 3 months	5 (11.3)	5.70 (5.70–7.00)	7.00 (4.00–13.00)
Stopped 3–6 months	9 (20.4)	6.80 (6.20–7.70)	13.00 (10.00–13.00)
Stopped 6–12 months	3 (6.8)	7.90 (7.30–8.35)	14.00 (11.50–15.50)
Stopped > 12 months	27 (61.3)	5.50 (5.30–7.00)	8.00 (6.00–11.50)
P-value†	44 (100.0)	0.281	0.198

† P-values derived from Kruskal–Wallis test.
ACS, Average Comet Score; IOD, Incidence of Damage.
Smoking history was self-reported. No statistically significant differences in SDF parameters were observed across smoking categories.
