# Supplementary material for: Double-stranded sperm DNA fragmentation measured with neutral comet assay as a predictor of IVF outcomes: evidence from three European clinics in a multi-centred prospective study
Source: Hum Reprod. 2026 Mar 28;41(5):677–88. doi: 10.1093/humrep/deag046 (PMC13139651; doi:10.1093/humrep/deag046)
Supplement: deag046_Supplementary_Table_S5 [file deag046_supplementary_table_s5.pdf]

Human Reproduction, 2026, 1–1
https://doi.org/10.1093/humrep/deag046
Supplementary Data

Supplementary Table S5. Sensitivity analyses adjusting for semen parameters.

Model	Additional covariates	dsSDF measure	Effect estimate (OR, 95% CI)	P-value
Primary model	Female age, male age, centre	ACS	0.84 (0.72–0.97)	0.025
Sensitivity model 1	+ Sperm concentration (million/ml)	ACS	0.84 (0.71–0.97)	0.025
Sensitivity model 1	+ Total motility (%)	ACS	0.84 (0.71–0.97)	0.025

Odds ratios (ORs) were derived from multivariable logistic regression models. Models were fitted separately for each dsSDF metric. The table is intended to demonstrate robustness of dsSDF associations after adjustment for selected semen parameters.
