# Supplementary material for: Double-stranded sperm DNA fragmentation measured with neutral comet assay as a predictor of IVF outcomes: evidence from three European clinics in a multi-centred prospective study
Source: Hum Reprod. 2026 Mar 28;41(5):677–88. doi: 10.1093/humrep/deag046 (PMC13139651; doi:10.1093/humrep/deag046)
Supplement: deag046_Supplementary_Table_S6 [file deag046_supplementary_table_s6.pdf]

OXFORD
human reproduction

Human Reproduction, 2026, 1–1
https://doi.org/10.1093/humrep/deag046
Supplementary Data

Supplementary Table S6. Sensitivity analyses adjusting for semen parameters.

Model	Additional covariates	dsSDF measure	Effect estimate (OR, 95% CI)	P-value
Primary model	Female age, male age, centre	IOD	0.95 (0.90–0.99)	0.025
Sensitivity model 1	+ Sperm concentration (million/ml)	IOD	0.95 (0.90–0.99)	0.025
Sensitivity model 1	+ Total motility (%)	IOD	0.95 (0.90–0.99)	0.025

Odds ratios (ORs) were derived from multivariable logistic regression models. Models were fitted separately for each dsSDF metric. The table is intended to demonstrate robustness of dsSDF associations after adjustment for selected semen parameters.

© The Author(s) 2026. Published by Oxford University Press on behalf of European Society of Human Reproduction and Embryology.
This is an Open Access article distributed under the terms of the Creative Commons Attribution License (https://creativecommons.org/licenses/by/4.0/), which permits unrestricted reuse, distribution, and reproduction in any medium, provided the original work is properly cited.
Human Reproduction, 2026, 00, 1–18
https://doi.org/10.1093/humrep/deag046
Original Article
